# Interdisciplinary Mobile Health Model to Improve Clinical Care After Heart Transplantation: Implementation Strategy Study

**DOI:** 10.2196/19065

**Published:** 2020-11-24

**Authors:** Mar Gomis-Pastor, Sonia Mirabet, Eulalia Roig, Laura Lopez, Vicens Brossa, Elisabeth Galvez-Tugas, Esther Rodriguez-Murphy, Anna Feliu, Gerardo Ontiveros, Francesc Garcia-Cuyàs, Albert Salazar, M Antonia Mangues

**Affiliations:** 1 Pharmacy Department Hospital de la Santa Creu i Sant Pau Barcelona Spain; 2 Heart Failure and Heart Transplant Unit Cardiology Department Hospital de la Santa Creu i Sant Pau Barcelona Spain; 3 Information System Department Hospital de la Santa Creu i Sant Pau Barcelona Spain; 4 Deputy Medical Director, Hospital Sant Joan de Déu Barcelona Spain; 5 Director Manager, Hospital Universitari Vall Hebron Barcelona Spain

**Keywords:** cardiology, heart transplantation, implementation strategy, health care model, integrated health care systems, interdisciplinary health team, medication therapy management, health care technology, mHealth, eHealth

## Abstract

**Background:**

Solid organ transplantation could be the only life-saving treatment for end-stage heart failure. Nevertheless, multimorbidity and polypharmacy remain major problems after heart transplant. A technology-based behavioral intervention model was established to improve clinical practice in a heart transplant outpatient setting. To support the new strategy, the mHeart app, a mobile health (mHealth) tool, was developed for use by patients and providers.

**Objective:**

The primary objective of this study was to describe the implementation of the mHeart model and to outline the main facilitators identified when conceiving an mHealth approach. The secondary objectives were to evaluate the barriers, benefits, and willingness to use mHealth services reported by heart transplant recipients and cardiology providers.

**Methods:**

This was an implementation strategy study directed by a multidisciplinary cardiology team conducted in four stages: design of the model and the software, development of the mHeart tool, interoperability among systems, and quality and security requirements. A mixed methods study design was applied combining a literature review, several surveys, interviews, and focus groups. The approach involved merging engineering and behavioral theory science. Participants were chronic-stage heart transplant recipients, patient associations, health providers, stakeholders, and diverse experts from the legal, data protection, and interoperability fields.

**Results:**

An interdisciplinary and patient-centered process was applied to obtain a comprehensive care model. The heart transplant recipients (N=135) included in the study confirmed they had access to smartphones (132/135, 97.7%) and were willing to use the mHeart system (132/135, 97.7%). Based on stakeholder agreement (>75%, N=26), the major priorities identified of the mHealth approach were to improve therapy management, patient empowerment, and patient-provider interactions. Stakeholder agreement on the barriers to implementing the system was weak (<75%). Establishing the new model posed several challenges to the multidisciplinary team in charge. The main factors that needed to be overcome were ensuring data confidentiality, reducing workload, minimizing the digital divide, and increasing interoperability. Experts from various fields, scientific societies, and patient associations were essential to meet the quality requirements and the model scalability.

**Conclusions:**

The mHeart model will be applicable in distinct clinical and research contexts, and may inspire other cardiology health providers to create innovative ways to deal with therapeutic complexity and multimorbidity through health care systems. Professionals and patients are willing to use such innovative mHealth programs. The facilitators and key strategies described were needed for success in the implementation of the new holistic theory–based mHealth strategy.

## Introduction

### Background

Solid organ transplantation could be the only life-saving treatment for end-stage heart failure [1]. Since the first heart transplant was performed in 1967, recipients’ life expectancy has markedly increased [2-4], making heart transplant a chronic condition. Nevertheless, the improvement in survival has been accompanied by greater multimorbidity [5,6] and long-term complexity [7-9]. Five years posttransplant, 95% of heart recipients have hypertension, 81% have hyperlipidemia, 33% have chronic renal failure, and 32% have diabetes [7]. In addition, nonadherence to lifestyle recommendations (eg, diet, exercise, or blood pressure monitoring) is frequent after transplant, with serious consequences for survival [10,11].

Another challenge for heart transplant recipients is the lifetime need to rigorously follow a regimen of immunosuppressive therapy to avoid rejection and to take multiple drugs to treat comorbidities [12]. Five years posttransplant, heart recipients take an average of 10 drugs [13], with a third of them taking more than 16 medications per day [8]. These therapeutic complexity rates are high compared with those in other chronically ill populations [8,9,14,15], increasing the risk of poor therapeutic adherence [16], pharmacological interactions and medication adverse effects [17-19], impaired quality of life [20,21], hospital readmissions [22], and even mortality [23]. In particular, 20%-50% of recipients are nonadherent to immunosuppressive treatment [24,25], which is worrisome owing to the association between nonadherence and graft failure, rejection, and poor survival after heart transplant [10,24].

The search for clinical improvement practices to deal with multimorbidity and polypharmacy is currently a priority for heart transplant providers [26]. Longer morbidity-free survival rates and enhanced quality of life [2,27] could be achieved by improvements in healthy lifestyle habits, medication management, and quality of care [2,11]. Some promising strategies have already been tested in clinical practice and are ready to be applied in the heart transplant population.

First, integrated and comprehensive health care programs carried out by proactive teams could enhance health outcomes [28,29]. Well-trained interdisciplinary teams have been associated with better management of chronicity after heart transplant [30,31]. Second, the use of internet-based (eHealth) systems, including web and mobile health (mHealth) apps, has been reported to improve lifestyle and medication management in chronic conditions [32-42]. According to the International Society for Research on Internet Interventions (ISRII) statements [43] and other authors [44], internet-based models are an opportunity to deliver interventions to produce a cognitive and behavioral change in patients. Such interventions consist of “treatments, typically behaviorally based, that are operationalized and transformed for delivery via the internet” [43,45-47]. To increase their efficacy, these interventions are typically tailored to an individual’s needs and environment, based on electronic patient-reported outcomes [36,40,48]. The establishment of new internet-based interventions in the field of transplantation is promising [26,49,50], but holistic models based on behavioral change technologies in heart transplant population are still scarce.

**Figure 1:**
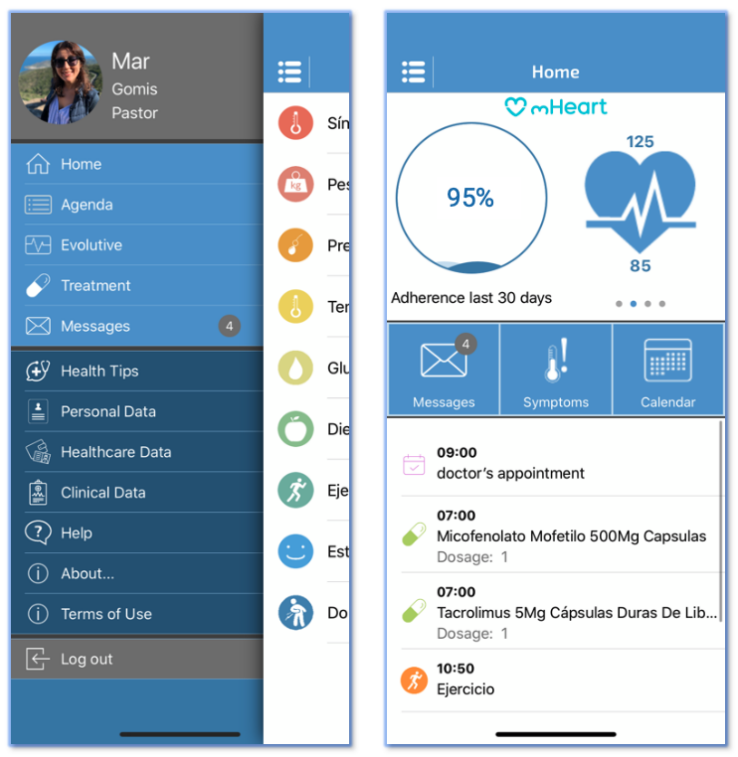
The mHeart system menu, displaying the different app modules: Treatment, Agenda, Self-control, Symptoms, Messaging, Health Education and Advice, Personal and Clinical Data.

Based on these strategies, an internet- and theory-based holistic intervention model was implemented for the first time in the heart transplant outpatient clinic of a tertiary hospital. The new practice was designed to help health providers improve medication safety and effectiveness, patient-provider interactions, and comprehensive clinical care. The tool created to support the interventional program, the mHeart system, was a mobile app complemented by a website for use by patients and providers (Figure 1). Establishing the new model was costly and time-consuming and its implementation in usual practice posed several challenges to the multidisciplinary team in charge. The skills of the health providers in charge, such as patient engagement, motivational interviewing, and management [51], were essential to lead the implementation. The mHeart system and the theory-based interventional health care program were designed to offer a solid starting point to improve health outcomes in complex populations such as heart transplant patients.

Outlining the methodology used, principal findings, and the barriers and facilitators encountered in usual clinical practice could be highly useful for new developers and could be generalizable in other contexts. Therefore, this article may guide other health providers in the implementation of holistic and interdisciplinary internet-based strategies to improve clinical practice.

### Objectives

The main objectives of this study were (1) to describe the implementation of a holistic interdisciplinary technology-based behavioral intervention model to improve therapy management and the clinical care of heart transplant recipients, and (2) to outline the facilitators for future implementations based on the experience gained.

Secondary objectives were to assess patients’ access to technology and willingness to use mHealth services, and to analyze stakeholders’ opinions of the major gains and barriers to an mHealth approach.

## Methods

### Study Design and Setting

This study is based on an implementation strategy of a clinical practice improvement model. The study was conducted in a heart transplant outpatient setting of a cardiology unit of a tertiary university hospital between 2014 and 2017. A mixed methods design was applied and included several surveys, interviews, and focus groups. The study was approved by the institutional review board (IIBSP-MHE-2014-55). Participants were adult outpatient heart transplant recipients; representatives of patient associations; health professionals; providers; and experts in quality, safety, or legal fields. Participants were informed of the study objectives and of the team conducting the study. All participants provided written consent.

The Standards for Reporting Implementation Studies [52] were followed for transparent and accurate data reporting for the entire study. When the content analysis method was used from group discussions, the Consolidated Criteria for Reporting Qualitative Research (COREQ) [53] were applied. In addition, the directions for the ISRII [43] and the CONSORT-EHEALTH guidelines [47] were followed to report the internet-based interventional program, as appropriate.

### Procedures

#### Phases and Team

**Figure 2:**
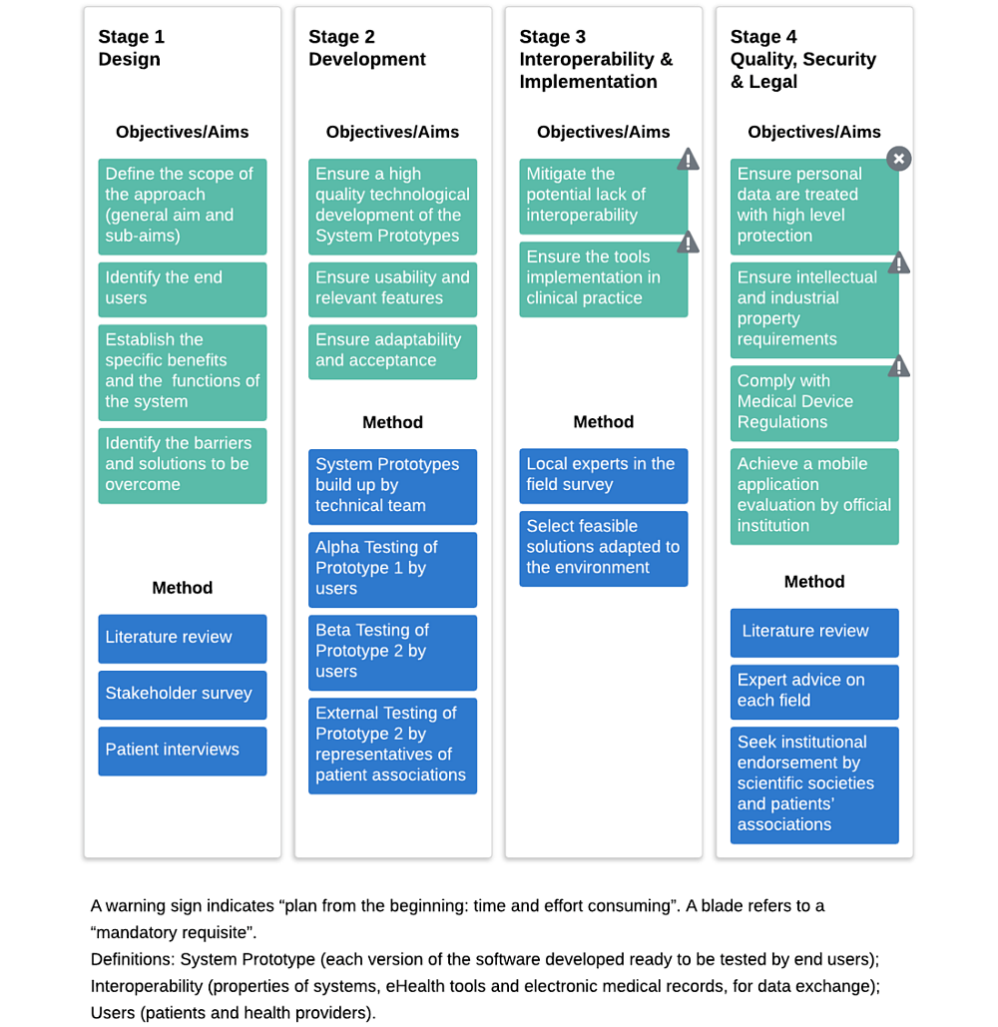
Summary of the procedures and stages followed during implementation of the mHeart approach.

The internet-based strategy was carried out in four stages, including design; development; interoperability and implementation; and quality, security, and legal requirements. A summary of the aims of the stages and the methodology used is provided in Figure 2.

The interdisciplinary clinical team in charge of the mHeart system was the hospital’s scientific advisory team, composed of 4 cardiologists, 2 cardiology nurses, 1 cardiology psychologist, and 2 clinical pharmacists. All of them were female except for the male cardiologist. Among the pharmacists, one was a transplant pharmacist with experience in motivational interviewing and transplant therapeutics, while the other had broad experience of managerial skills. The transplant pharmacist was assigned as the scientific coordinator and undertook the following tasks: facilitating procedures and meeting deadlines, prioritizing tasks, liaising with participants and the technical team, and reporting to the scientific advisory team.

#### Stage 1: Design

Stage 1 lasted from April 2, 2014 to March 15, 2015. During this period, distinct methodologies were combined to establish the following approach.

First, the software was categorized by the scientific advisory team as a behavior intervention technology to facilitate relevant overall goals: health behavior change (ie, increase patients’ healthy behaviors and prevent the onset of disease) and targeted disease management (ie, facilitate therapeutic interventions and improve patients’ self-management). The system was initially conceived of as an mHealth software based on a mobile app for heart transplant recipients in the outpatient setting. The software was interactive with additional human support (ie, a multidisciplinary heart transplant team) [43]; thus, a website was also designed for providers.

Second, the scientific advisory team reviewed design models for the development of behavior intervention technologies, mainly that of Mohr et al [54] but also several others [55-58], which served as a guide for how to combine technology engineering with behavioral science. Several expert reports on the efficacy of internet-based interventions and system engagement were also reviewed [35,43,45-47,59-61]. Behavior change theories were used as a framework to design the interventions and software components. The interventional program was based on human support, motivational engagement, and therapeutic alliance [62,63]. The strategies applied included tailored feedback, among others [44,64-67]. The taxonomy of Abraham and Michie [68] was used to standardize the theory-based interventions in terms of discrete techniques. These techniques are fully described to improve the future replication of the approach and its adoption in usual clinical practice or research (Multimedia Appendix 1). Interactive elements were also used as digital triggers to prevent the law of attrition in eHealth interventions (eg, alerts, prompts, reminders, notifications, messages, and video calls) [62,69]. The components of the system aimed to deliver personalized interventions using motivational interviewing techniques, according to common practice in heart transplant centers [25,70].

Third, the scientific advisory team performed a literature review to guide the specific clinical subaims and software functionalities that should be prioritized in the model [54] and identify the barriers to be overcome, including institutional reports such as those of the US Food and Drug Administration, European Union, and Pharmacist Associations statements about eHealth [37-39,71-77]; studies on improving polypharmacy and chronic disease management [34,36,40-42,48,78,79]; and studies or reports describing patient-reported outcome measures with an impact on survival in heart transplant [7,10,11,79-85].

Fourth, the opinions of end users (ie, providers and patients) were evaluated. To assess patients’ access to technology and willingness to use mHealth services, the scientific coordinator performed a 45-minute, in-depth face-to-face interview with each adult chronic-stage (>1.5 years from heart transplant) recipient included in the study. The recipients were recruited consecutively in the Cardiology Outpatient Clinic from April 15, 2014 until April 2, 2015. The interviews aimed to determine patients’ current access, knowledge, and use of technology and their willingness to use an mHealth approach. The interview was based on a questionnaire previously reported by McGillicuddy et al [86]. Sociodemographic and clinical variables were collected from the patients’ electronic health records. The data collection sheet is provided in Multimedia Appendix 2.

To assess the stakeholders’ agreement about the gains and barriers associated with an mHealth approach in the heart transplant population, the scientific coordinator invited a purposive sample of stakeholders to participate in a survey. The themes were previously identified in the literature review and were related with benefits and barriers associated with an mHealth approach directed to multimorbid patients with polypharmacy (Multimedia Appendix 2). The survey was sent by email on September 29, 2014. The results were used to indicate which clinical subaims of the approach should be prioritized and the software design solutions necessary to overcome the limitations identified. An agreement of >75% of the stakeholders was considered adequate [87].

The following stakeholders were eligible for selection: interdisciplinary transplant staff (n=21), with no distinction being made in terms of age, knowledge of technologies, or favorable or unfavorable personal opinions about eHealth programs; technology analysts (n=2); experts in mHealth (n=3) (ie, the Regional Health Department specialist in innovative health care projects, the manager of the mHealth.cat Regional Health Department, and the Director of the mHealth Competence Center at Mobile World Capital); the hospital manager (n=1); and the manager of the Regional Technology, Innovation, and Public Health Department (n=1).

#### Stage 2: Development

Stage 2 lasted from March 15, 2015 to June 2, 2016 and aimed to design the technology and to test mHeart. The development of the system was assigned to a health care system apps firm. The technical team consisted of 1 analyst, 5 developers (superior systems engineers), 1 designer, and 1 project leader. The scientific coordinator intervened throughout the process, providing advice to the technical team and consulting with other providers when necessary. Development and testing environments were used by the technical team to respectively produce and consolidate the system prototypes before end users were involved. First, a general software structure was set up (mHealthCare system) to then direct it to heart transplant specifications and obtain the mHeart tool. The system was built as three apps: web, Android, and iOS mobile apps. To increase the scalability of the approach and data transparency, an in-depth description of the system’s technical details, the source code, and other relevant details are provided in an online dataset [88].

The mHeart prototypes were tested by end users in a Staging environment (alpha testing), followed by a Production environment (beta testing).

Alpha testing of Prototype 1 was performed to explore three domains: feature intuitiveness, esthetics, and new software elements or functions not considered during the design stage. With this aim, two distinct group sessions were held on September 15, 2015: one with the hospital’s scientific advisory team (n=9) and the other with heart transplant recipient volunteers consecutively recruited from the Cardiology Outpatient Clinic (n=6). Each session lasted 3 hours and was led by the technical team and the scientific coordinator. A video of the prototype was played to guide the groups through each of the prototype modules and functions. Participants were then asked to complete the same tasks using the tool on their smartphones. Software usability issues, uncompleted tasks, and doubts arising during the sessions were noted. At the end of the session, the three domains were explored. Field notes were recorded by a nurse of the scientific advisory team during the session. Conclusions were provided to participants at the end of the session for comments or corrections.

Beta testing of Prototype 2 aimed to obtain user feedback simulating a real-world home-based 4-week follow-up (January 10, 2016 to February 10, 2016). Participants consisted of the scientific advisory team (n=9) and volunteer heart transplant recipients consecutively recruited from the Cardiology Outpatient Clinic (n=6). Each day, participants electronically completed a data collection sheet with the following domains: technical issues, amendments suggested by the participants, and additional features not included in the prototype. The test findings were analyzed by the scientific coordinator in consensus with the technical team to prioritize tasks.

Additionally, an external session was held in the offices of the local transplant organization on October 25, 2016. Participants consisted of representatives of patient associations (n=7) recruited via telephone by the organization. The scientific coordinator conducted a 2-hour session with a video demonstration of prototype 2. The participants were then asked to complete the same tasks using the tool on their smartphones. At the end of the session, the domains explored were the tool’s acceptance, adaptability of the approach to other heart transplant centers, and any new queries or opinions. Field notes were recorded by a nurse of the scientific advisory team during the session. Conclusions were provided to participants at the end for comments or corrections.

#### Stage 3: Interoperability and Implementation

Stage 3 aimed to mitigate the potential lack of interoperability (the property of systems such as mHeart and medical records to exchange data) and to ensure the implementation of the approach in clinical practice. The survey designed is provided in Multimedia Appendix 2. Themes were identified in advance, including the available technical possibilities and resources to automatically transfer patients’ sociodemographic data from electronic health records to mHeart, and to upload data recorded in mHeart to medical records. Purposive participants were recruited by phone by the scientific coordinator: these participants consisted of the manager of the Hospital Information Analysis Department and the manager of the mHealth.cat Regional Health Department. The survey was sent by email on February 16, 2016. The responses were analyzed, and feasible solutions were prioritized by the scientific coordinator in consensus with the technical team.

#### Stage 4: Quality, Security, and Legal Requirements

Stage 4 aimed to ensure the quality and security of the internet-based platform. The scientific coordinator sought the involvement of hospital experts or external consultation on the following domains: data protection and confidentiality policy (n=2), legal requirements (n=2), intellectual and industrial property (n=3) and an external consultant (n=1), and evaluation of mobile apps standards and certifications (n=1). Feasible solutions were applied based on the experts’ requirements and technical possibilities. Finally, written endorsement of the quality content was requested from 1 regional health institution, 2 scientific societies, and 2 patient associations.

### Data Recording and Statistical Analysis

To ensure data accuracy, data collected during the study stages were recorded electronically in the online database Clinapsis [89] by a pharmacist. A second review was independently performed by a pharmacist and a physician. None of the data coders was part of the hospital’s scientific advisory team.

Statistical analysis was applied to analyze the results of patient interviews and stakeholder surveys. Categorical variables are reported as number and percentage. Quantitative variables are expressed as the mean and standard deviation. Nonnormally distributed variables are expressed as the median and interquartile range. The statistical analysis was performed with IBM SPSS (V22.0).

## Results

### Stage 1: Design

Regarding patient access to technology and willingness to use mHealth services, of the 158 recipients >1.5 years from heart transplant, 142 (89.9%) were assessed for eligibility and 135 (85.4%) were finally recruited and analyzed. Of the patients excluded, 5 were followed up in another transplant center, 5 had cognitive impairment, and 6 were palliative. Of the 7 recipients who declined to participate, the reasons were lack of interest (n=2), lack of time to complete the interview (n=4), and feeling too unwell to complete the interview (n=1).

Basic demographic and clinical data of the 135 chronic-stage heart transplant recipients interviewed are provided in Table 1. Briefly, the recipients’ mean age was 57 (SD 14) years and 31% were women. The mean time since transplant was 12 (SD 7, range 2-31) years and was ≥15 years in 32% of the sample. The mean total number of drugs prescribed was 12 (SD 3, range 5-21) to treat 6 (SD 3, range 0-11) comorbidities posttransplant.

Respondents’ access to technology and willingness to use mHealth services are described in Table 2. Patients’ opinions led to the inclusion of the following elements: the figure of the tutor (a caregiver or a close family member), a proactive technical support service, and a website profile for patients to complement the initial mHealth system.

**Table 1:** Chronic heart transplant recipients’ (>1.5 years from transplant) sociodemographic and clinical characteristics (N=135).

Variable		Value
Women, n (%)		41 (30.4)
Age at time of study inclusion (years), mean (SD)		57 (14)
**Time since transplant at the time of study inclusion (years)**		
	Whole sample, mean (SD), range	12 (7), 2-31
	>1.5-3, n (%)	11 (8.1)
	3-5, n (%)	16 (11.9)
	5-10, n(%)	27 (20.0)
	10-15, n (%)	37 (27.4)
	≥15, n (%)	43 (31.9)
BMI (kg/m^2^), mean (SD)		27 (6)
**Heart failure etiology, n (%)**		
	Coronary/ischemic	36 (26.7)
	Cardiomyopathy	58 (43.0)
	Other	41 (30.4)
Urgent heart transplant, n (%)		33 (24.4)
**Educational attainment, n (%)**		
	No schooling	15 (11.1)
	Middle school graduate	58 (43.0)
	High school graduate	25 (18.5)
	University graduate	36 (26.7)
**Employment status, n (%)**		
	Disability	74 (54.8)
	Retired	20 (14.8)
	No previous employment	7 (5.2)
	Currently working	33 (24.4)
Need or requirement for caregiver, n (%)		28 (20.7)
Lives with someone else, n (%)		115 (85.2)
Number of comorbidities, mean (SD), range		6 (3), 0-11
**Patients with comorbidity posttransplant, n (%)**		
	High blood pressure	94 (69.6)
	Dyslipidemia	73 (54.1)
	Chronic kidney failure	58 (50.0)
	Osteopathies and chondroplasties	52 (38.5)
	Diseases of the nervous system	51 (37.8)
	Mood and anxiety disorders	49 (36.3)
	Digestive system diseases or disorders	42 (31.1)
	Diabetes mellitus	42 (31.1)
	Neoplasia	39 (28.9)
	Arthropathies	27 (20.0)
Total number of drugs prescribed, mean (SD); range (IQR)		12 (3); 5-21 (9-14)

**Table 2:** Chronic heart transplant recipients’ (>1.5 years from transplant) access to technology and willingness to use mobile health (mHealth) services (N=135).

Variable		Value
Number of devices per patient, mean (SD)		2.2 (0.7)
**Types of devices owned by patients, n (%)**		
	Mobile phone	132 (97.8)
	Computer	98 (72.6)
	Tablet	60 (44.4)
**Internet access on patients’ devices, n (%)**		
	3G or 4G connection	112 (83.0)
	Only connects to the internet using WiFi	18 (13.3)
	Does not know/no response	5 (3.7)
**Frequency of technology use, n (%)**		
	Often	87 (64.4)
	Sporadically	35 (25.9)
	Never	13 (9.6)
**Internet usage for health-related purposes, n (%)**		
	Often	41 (30.4)
	Sporadically	43 (31.9)
	Never	51 (28.1)
**Initial assessment of the mHealth approach, n (%)**		
	Not very useful	2 (1.5)
	Useful	92 (68.1)
	Very useful	40 (29.6)
	Not yet known until the platform is tested	1 (0.7)
**Initial assessment of mHeart type of platform, n (%) (multiple choice)**		
	Interested in using mHeart mobile app	81 (60.0)
	Interested in using mHeart website	64 (47.4)
	Not yet known until the platform is tested	40 (29.6)
Initially requires a tutor to use the platform, n (%)		30 (22.2)

According to stakeholder agreement about the benefits and barriers of an mHealth approach, of the 31 stakeholders invited to complete the survey, 2 nurses, 2 cardiologists, and 1 social worker did not respond. No reasons were reported. Finally, 26 stakeholders responded to the questionnaire, 17 (65%) were women with a mean age of 46 (SD 10) years. The profiles of the 26 participants were: 6 (23%) physicians, 3 (11%) nurses, 5 (19%) pharmacists, 2 (8%) psychologists, 2 (8%) technology analysts, 3 (11%) key representatives of local health authorities, 2 (8%) representatives of regional health authorities, and 3 (12%) experts in mHealth.

The main gains of the mHeart strategy according to stakeholders’ opinions are detailed in Table 3. Consensus was strong for the use of mHealth to improve therapy management (>85%). In this sense, the mHeart key features were mainly designed according to the aims presented in Textbox 1. Strong agreement (>75%) was also achieved for several other comprehensive benefits. Thus, the software features design was also directed to promote patient-provider interactions and communication, and to empower patients to play a more active role in their lifestyle, treatment, and self-care. The major barriers of an mHealth approach identified by stakeholders are described in Table 3. Of note, agreement among stakeholders was weak for all items (<75%). Relevant barriers were prioritized to be overcome by the hospital’s scientific advisory team due to their impact on implementation and scalability: (1) ensuring the system’s legal requirements, quality, and data security; (2) mitigating end users’ digital divide (providers and patients); (3) achieving system interoperability; and (4) building the mHeart software in a global structure that could be easily adapted to other complex diseases.

**Table 3:** Stakeholders’ agreement on the benefits and limitations of a mobile health approach in multimorbid patients with polypharmacy such as the heart transplant population (N=26).

Statement for agreement		Stakeholders, n (%)
**Benefits**		
	Improves patients’ knowledge of therapy, management, and medication adherence	23 (88)
	Improves the continuity of care and the flow of information between providers and levels of care	21 (81)
	Allows patients to be empowered and actively manage their disease and treatment	20 (77)
	Resolves patient and caregiver queries from home due to the two-way health care provider-patient communication	20 (77)
	Monitoring and managing patient-reported outcomes such as symptoms and adverse effects to drugs	17 (65)
	Focuses on health promotion and prevention to reduce the number of acute events	17 (65)
	Increases the cost-effectiveness of resources by reducing both scheduled and urgent visits due to decompensation	17 (65)
	Facilitates innovation in health and documentation of evidence that translates into measurable health outcomes	17 (65)
	Reduces inequalities in access to the health system due to traveling difficulties or lack of resources	10 (38)
	Improves patients’ experience because of close communication with providers	4 (15)
**Limitations**		
	Increase in workload for staff	15 (58)
	Lack of institutional guidelines to set up and implement systems and accreditation of mobile health apps	14 (54)
	Risk of not sharing the patient’s registered information with other levels of care or with other apps (used to manage other health conditions)	13 (50)
	Risk of not protecting confidential patient data	6 (23)
	Risk of creating inequalities in patient care due to resistance to use technology or the digital divide	6 (23)
	Lack of guarantee of the long-term economic sustainability of research projects for innovative technologies and companies that develop the systems	4 (15)

**Main aims of the mHeart strategy and software according to stakeholder’s agreement.**

- Improve therapy management (>85%)
  Identify nonadherent patients and determinants of medication nonadherence.
  Identify potential pharmacological interactions and adverse effects.
  Improve patients’ knowledge and management of regimens.
  Reinforce patients’ coresponsibility in their treatment.
  To provide early medication adjustments and tailored interventions based on patient-reported outcomes.
- To promote patient-provider interactions and communication (>75%)
- To empower patients to play a more active role in their lifestyle, treatment, and self-care (>75%)

### Stage 2: Development

As a result of the alpha testing with focus groups, additional features and improvements in functionality were implemented; the list is fully detailed in Multimedia Appendix 3. Beta testing feedback greatly improved usability, and the suggestions not affecting usability or security were postponed to subsequent mHeart improvement phases. New developers could incorporate these challenges described in Textbox 2 into their initial design of the system.

**Beta testing suggestions postponed to subsequent mHeart improvement phases; new developers could incorporate these challenges into the initial design of a new system.**

- Automatic responses to consultations regarding interactions with concomitant therapies connected to the official database.
- Programming periodic changes to the mHeart questionnaire type or order of items (eg, adherence or general condition). This will prevent the patient from responding in a routine manner and the system from losing sensitivity in identifying nonadherent patients.
- Set up a discussion forum for patients.
- Enable patients at home to print the medication chart and the calendar with all tasks planned in the tool’s agenda by providers and patients.
- Connecting the mHeart agenda with the hospital visit scheduling system to automatically download the appointment schedules on the mHeart system.
- Develop a decision support system based on artificial intelligence algorithms (patterns and prediction rules).
- Translating the platform into other languages to make the tool usable in other countries.

Important contributions were also obtained from patient association opinions. First, participants showed interest in using mHealth to manage their chronic comorbidities. Moreover, they highlighted their interest in two-way messaging with the clinical team. Participants also compared the tool with other free downloadable tools from online stores. Thus, the main additional value of mHeart noticed by the participants was primarily that it was adapted to their condition by transplant providers and that they could obtain clinical feedback on the activity recorded. Finally, they requested a patients’ chat room and a patient-provider teleconference module.

**Figure 3:**
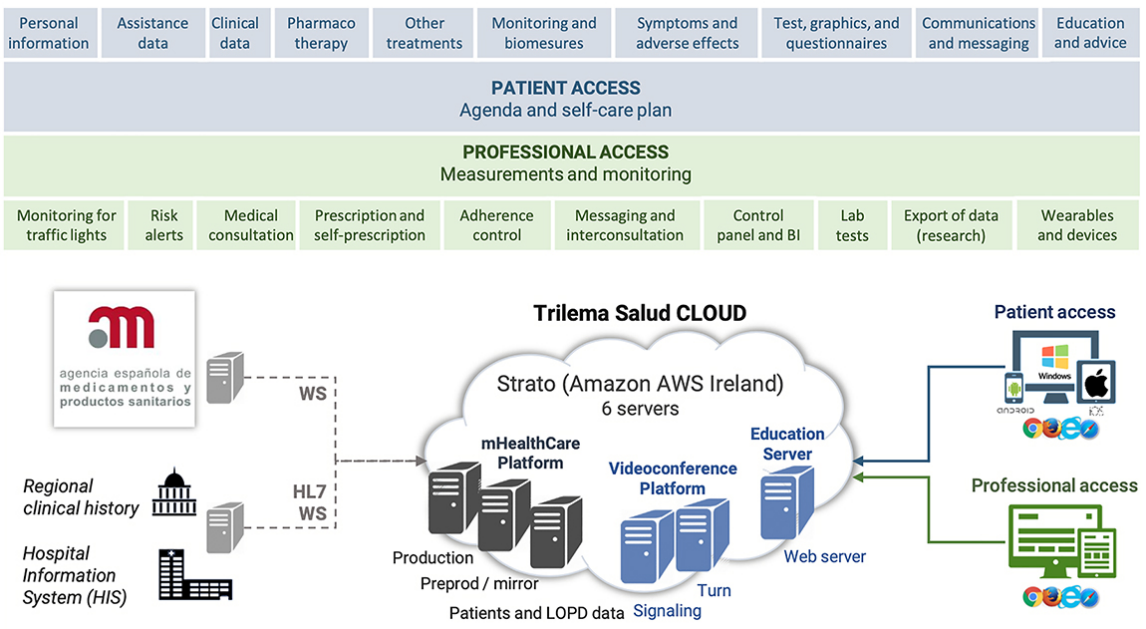
Functional layers and cloud architecture of mHeart. HIS: hospital information system; LOPD: the Spanish Organic Data Protection Law; WS: web server; HL7: High Level-7.

The entire technical development and user testing processes resulted in the final prototype of mHeart primarily directed to carry out integral therapy management and clinical care in transplant populations, and specifically in heart transplant recipients. The system is a mobile phone app connected to a website [90] for use by providers and patients. The app can be downloaded free from the online Google [91] and Apple [92] stores. The general layout is represented in Figure 3 and is detailed in the online dataset [88]. From a clinical point of view, the tool can be simultaneously used on distinct devices to facilitate support from caregivers or tutors. The use of the platform by patients and the multidisciplinary team are summarized in Tables 4 and 5. The behavioral framework and theory-based interventions that could be delivered using the mHeart tool in future intervention studies are listed in Multimedia Appendix 1. More details about functionalities and a video of the clinical use of the mHeart mobile app are also provided in the online dataset [88].

**Table 4:** mHeart patient profile modules, components, and clinical use.

Patient module	Components and clinical use
Treatment	Medication list including information on inactive drugs. Enquire about interactions consultation (ie, ask transplant pharmacist about new therapies).
Patient-Centered Module	Consulting and recording data (manually or using wearables). Reminders can be scheduled in the Agenda module. (1) Vital signs (ie, blood pressure, temperature, pulse and respiratory rate) and biomeasurements (ie, weight, height, glycemia). (2) Dietary intake, exercise data, and general wellness. Health instruments: adherence to medication (Haynes-Sachet [94] and Morisky-Green 4-item scale [95]), insomnia (Insomnia Severity Index [96]), and quality of life (EQ-5D-3L [97]). (3) Symptoms or adverse effects. The symptoms connected with an alert to clinicians were diarrhea, vomiting, fever, fainting episode, and syncope.
Agenda	The content of diverse modules is uploaded. A Push text alert can be activated on the patient’s mobile phone. (1) Medication timing and consultation of recommendations. (2) Drug intake recording (single or several drugs at the same time) and reasons for nonadherence (drop-down list). (3) Nonpharmacological prescriptions (eg, relaxation practice according to the psychologist’s prescription). (4) Tasks from the Patient-Centered Module programmed (eg, blood pressure monitoring 3 times per week). (5) Health reminders (eg, appointments, blood tests).
Communication Aids	(1) Teleconference: individual and group sessions. (2) A private patient-provider chat. Files can be attached.
Health Advice	Healthy lifestyle and health promotion information (eg, texts, photographs, or multimedia files).
Personal and Clinical Data	Sociodemographic data, documented allergies, and provider profiles (including affiliation and picture).
Help	(1) A help center service to solve both technical and functional problems (ie, telephone number, private message, and email). (2) Clinical contact data: medical team, pharmacist, transplant coordinator, patient appointment center, etc.
About	Information about the developers, aim of the tool, and team in charge.
Terms of Use and Privacy Policy	All the legal requirements already accepted should always be available for consultation.

**Table 5:** mHeart professional profile modules, components, and clinical use.

Provider Module	Component and clinical use
Patient View	List of active patient filters to organize the list and perform a rapid search.
Patient Registration	(1) The center identification number is used to download patient data from the hospital information system. (2) The patient receives a private message with login credentials. (3) Providers individualize the patient-reported outcome measures, schedule, and the treatment plan and recommendations for each new patient.
Treatment Prescription	(1) Pharmacological treatment is prescribed from a drop-down list of drugs updated from the Spanish National Formulary. Tailored recommendations can be added (eg, “Antirejection treatment. It is recommended that you take this on an empty stomach”). (2) Nonpharmacological therapies can be prescribed in free-form data entry by the multidisciplinary team (eg, nonsalty diet).
Patient-Centered Data Consultation	All data recorded in the Patient-Centered module can be tracked graphically in tables and diagrams. Timeframe filters can be used. mHeart platform features designed to follow medication adherence are adherence test results and drug intake registrations: (1) A traffic light system of alerts indicating a decrease in the patient’s weekly adherence. List of patients can be sorted by adherence rate to prioritize interventions. (2) Adherence rates are presented graphically and through tables (for each drug and for the overall treatment).
Communication Aids	(1) Individual patient-provider chat. (2) Group messaging. Filters are available. Large-scale interventions can be scheduled (eg, preventive health promotions) for specific time periods. (3) Teleconsultation patient(s)-provider(s) for individual or group visit. (4) Teleconference for interdisciplinary communication and shared decision-making between providers.

### Stage 3: Prototype Interoperability and Implementation

Diverse solutions to address implementation were settled by the scientific advisory team. First, mHeart was set up to be compatible with different systems and apps to ensure that users could employ their own phones, computers, or tablets. Second, technical support was outsourced (by the technological development firm) to provide initial training on mHeart skills to patients and providers as well as to solve queries. Finally, institutional protocols were created to standardize the new clinical workflows.

Additionally, based on participants’ expertise (n=2, 100%), the pathways to overcome the lack of integration and communication between mHeart and electronic health records were separated into local and institutional solutions. Regarding local solutions, the strategies embedded allowed for two-way data exchange between mHeart and the hospital information system. First, the mHeart system requests sociodemographic patient data from the hospital information system. Data can refer to a new patient or an update on the patient’s data. This is achieved via a synchronous high level-7 message patient query through the Simple Object Access Protocol. Second, once a week, a data report containing all of the mHeart patient-reported outcome measures is uploaded to the hospital information system. This is achieved via an implicit File Transfer Protocol over the Transport Layer Security server. A security process identifies the report and assigns it to the patient in the hospital information system. Only the latest report can be consulted as a clinical document. More details are also provided in the online dataset [88].

According to institutional solutions identified, the patient’s data report could also be integrated with the regional electronic clinical record. With this report, any provider in the catchment area can monitor patients from any care level (eg, primary care, hospital care). In addition, in 2017, the regional health care system approved mHeart to be integrated with La Meva Salut, which is a patient health website allowing citizens to interact with the regional health care system.

### Stage 4: Quality, Security, and Legal Requirements

Based on expert feedback, workable solutions were identified (listed in Textbox 3) to ensure legal, security, and data protection; medical technology intellectual property; medical device regulations; and quality evaluation. The solutions embedded could be used by other developers as a checklist to ensure minimum standards but are not limited to these solutions.

**Workable solutions to ensure the quality and security of the eHealth platform.**

**Processing personal data with confidentiality and security**

- Comply with the national regulations on high-level confidential personal data.
- Obtain support from the hospital’s Department of Data Confidentiality and Data Analysis.
- Ensure the quality of the Data Center through certification.
- Use secure connections for data integration between systems.
- Perform an annual audit of confidentiality and security by an external firm.
- Ensure users’ duties: (1) patients should sign a nondisclosure agreement; (2) passwords require updating every 6 months; (3) acceptance of mHeart’s conditions of use is a prerequisite and should always be available for future consultation by users.

**Intellectual and industrial property recommendations**

- Obtain support from experts on medical technology intellectual and industrial property.
- Sign a collaboration contract between the hospital and the developer’s private firm.
- Register the platform trademark (eg, “mHeart”).
- Register the platform content on intellectual property registers.

**Medical device certificate**

- Adopt the legislation requirements on medical device regulations [74,97]. CE marking as a class IIa medical device was obtained for mHeart.

**Certification granted by a local institution**

- Certificate of app quality by local institutions. AppSaludable [98] is already adopted for mHeart. AppSalut [99] is in the process of adoption by Fundació TicSalut (Regional Health Department). Some other options are British [100,101], iSYS Score [102], and uMARS [103,104].

**Content quality**

- Obtain institutional endorsement by scientific societies related to the population field. Written support for mHeart was provided by:
  - The regional transplant organization (OCATT) (October 31, 2016).
  - The regional transplant society (SCT) (October 10, 2017).
  - La Meva Salut homologation approval by the regional Health Government (October 20, 2016).
- Obtain written endorsement from patient associations and support groups. Written support for mHeart was provided by:
  - “Club de la Cremallera” Clinic Hospital (November 3, 2016).
  - “Cors Nous” Bellvitge Hospital (November 3, 2016).

## Discussion

### Principal Findings

The steps and key literature outlined in this paper resulted in the implementation of a holistic internet- and theory-based intervention model for the heart transplant population in the outpatient setting. After design of the mHeart system, several time-consuming issues remained to be resolved, such as interoperability, implementation, security, and quality. Moreover, the involvement of the interdisciplinary team, patients, and several experts was essential for the success of the platform but also required complex interactions.

Scalable, interactive apps directed to improve clinical practice are costly and time-consuming to produce [43]. We found several potential barriers when implementing the internet-based program in multimorbid patients, which are well known to lead to “dead ends” in real-world clinical practice [36,39,77]. Based on the experience gathered, the key points deemed essential in conceiving a new behavioral interventional model are outlined in Textbox 4. These recommendations could be used by future developers as a checklist to ensure minimum standards.

**Key recommendations for successful implementation of new eHealth strategies for new developers.**

1. Avoid new developments from scratch. Tools that are already established and tested are an efficient starting point. This will help to allocate the economic resources on new features, facilitating the meeting of deadlines and achieving the expected quality of the system.
2. Before choosing the development company, determine that (i) it is a solvent and solid firm, (ii) its compliance with national standards of quality and safety, (iii) it has previous experience of clinically tested health care systems, (iv) it has favorable opinions of previous developers, and (v) it provides an excellent user help center.
3. Allocate resources to having expert advice on (i) legal, security, and data protection; (ii) medical technology intellectual property; and (iii) medical device regulations and quality evaluation.
4. Assign a provider as a part-time coordinator to facilitate procedures and deadlines, and to liaise with third parties. The recommended skills of the coordinator are a proactive approach; holistic vision; experience of research and innovative projects; ability to work in a team; and to have training in a specialty, medication management, behavioral change theories, and patient engagement.
5. First, design a general system structure and later adapt it to the target population needs. This will help to ensure end-user engagement while compensating for the implementation burden and ensuring the scalability of the model.
6. Base the design of the interventional model on already demonstrated major determinants of the efficacy of interventions and patient engagement: (i) proactive and trained multidisciplinary teams, (ii) active interaction with end users, (iii) behavioral change theories, and (iv) tailored interventions based on relevant patient-reported outcome measures.
7. Include in the design stage: (i) an analysis of end users’ expectations, fears, and barriers; (ii) expert opinions on the interoperability of the system; and (iii) a plan for sustainability and reimbursement according to the interests of the center or health institution.
8. Join forces with patient associations and scientific societies during the design and testing stages to ensure content quality and scalability among centers.
9. Evaluate whether new features that may arise in the testing are (i) incorporated in the prototype (only recommended if they affect the usability and quality of the system), or (ii) addressed in subsequent phases of improvements.
10. Once the final prototype is established, resources should be allocated to provide continuous updates based on users’ needs and feedback. This will ensure the system’s usability, quality, and persistence over time.

### Barriers and Facilitators to Implementing the mHeart mHealth Approach

Consideration of the issues to overcome during the implementation of mHeart could shorten the time period to reach the desired quality standards. Thus, it is critical for any new development to be based on an in-depth analysis of feasible solutions to overcome limitations. The first potential barrier to implementing an mHealth solution according to the opinion of 58% of the stakeholders was the *increase in clinicians workload*. However, in line with previous studies [36,45,105], the burden experienced during mHeart implementation was mainly derived from several other reasons such as achieving a well-designed theory-based framework of the intervention model, ensuring legal and security requirements, involving the health care team in training and workflow, and, ultimately, several organizational barriers. These tasks were highly demanding of time, and therefore it is strongly recommended that future developers perform an initial roadmap based on successful previous experiences. Moreover, an initial agreement with all of the parties involved on the stages and their responsibilities is also critical to reduce burden.

The second most widely agreed barrier, by 50% of respondents, was *lack of interoperability*, which has also been identified by other authors [33,39,77] as a major risk factor for unsuccessful eHealth approaches becoming isolated from the health care system. This challenge was technically demanding, but entails improvements in safety and quality. Indeed, mHeart testing of interoperability revealed that transcription errors could be avoided, the time spent typing patient data decreased, and better coordination among providers could be achieved.

Other well-established major barriers of eHealth strategies in clinical practice [72], and in line with respondents’ opinions, were the *lack of models for funding* (15%) and *reimbursement for mHealth services by health systems* (54%). Although local guidance is fortunately growing [39,106], there is a delay in the implementation of new telemedicine laws [51]. This causes uncertainty about minimum quality standards and hinders scalability because of a lack of reimbursement models [38,73,107]. The initial mHeart funding was based on grants and has been detailed in the online dataset [88] to increase transparency and inspire new developers to overcome this barrier.

The risk of *patient’s* *resistance to using technology or the digital divide* was also a potential barrier according to 23% of the stakeholders, and is in agreement with a previous finding in multimorbid patients [33]. Nevertheless, almost all of the recipients in this study owned a cell phone and agreed on the utility of mHealth approaches such as mHeart. Thus, these data reinforce the idea of access, widespread use, and acceptance of technology in the heart transplant population, as previously observed in transplant recipients [86,108]. Nevertheless, high levels of attrition are a real issue in eHealth programs [62]. Thus, a persuasive design focused on enhancing user adherence is highly recommended [69,109,110]. Moreover, patients’ opinions should also be carefully considered, with special emphasis on identifying potential barriers. In the mHeart interviews, up to 47% of recipients were interested in using a complementary website and 22% reported the need for a tutor to use the tool. Thus, a patient profile website was provided, and a help center was hired to provide human assistance and initial training to users; according to other authors [62], this strategy has potential to increase user engagement without increasing provider burden.

### Benefits of the mHeart Strategy in Multimorbid and Polypharmacy Populations Such As Heart Transplant Recipients

The information gathered from the opinions of patients and stakeholders allowed us to establish the aims of the mHeart clinical practice improvement model. Thus, the theoretical gains of mHealth described in the literature were translated into real-world strengths and the key software features were designed to achieve them. First, the *improvement in medication safety and efficacy* achieved the highest agreement by the stakeholders surveyed (88%), which supports previous studies [111-113] highlighting safety and efficacy as a major determinant in health outcomes. Thus, the main feature of mHeart was to provide pharmaceutical care, with particular emphasis on reducing the impact observed [10,24] of nonadherence to immunosuppressants after transplant. To succeed, the mHeart design combined multilevel strategies inspired by previous successful experiences [38,114,115], including educational, motivational, and tailored internet theory–based interventions to be delivered by a proactive team [12,25,41].

The two main strengths of the mHealth approach were *improving continuity of care and information flow* (81%) and *solving patient and caregiver queries* (77%). Indeed, based on the opinions of patient association representatives and in line with the findings of other authors [33,111], chronic patients are seeking more communication opportunities and better coordination among providers. In this sense, mHealth programs represent a unique opportunity to combine human support and new digital skills to reach a therapeutic alliance with the patient [109,110]. Software functions to promote patient-professional interaction [62,69] are therefore essential in a patient-centered model such as mHeart targeting the outpatient population.

Other relevant gains of mHealth reported by stakeholders were *enhancing patient’s self-management* (77%), *early detection of symptoms or adverse effects* (65%), and *the use of patient-reported outcomes to allow preventive strategies* (65%). Indeed, the current scenario, in which patients are demanding coresponsibility [63], provides a strong opportunity to engage patients in electronically recorded patient-reported outcomes but also to train them in how to detect alarm symptoms and how to act when they arise. The use of patient-reported outcomes has previously shown an impact on medication efficacy and safety [36], patients’ quality of life, and even survival [40]. Thus, it is expected that preventive internet-based interventions based on patient-reported outcomes will be a determinant to improve outcomes in outpatient care in the near future.

### Opportunities Derived From Implementation of the mHeart Model

Successful eHealth interventions are commonly directed to specific population needs, such as mHeart in the heart transplant population [45,116]. This was indeed a particular strength highlighted by the patient associations during the testing of mHeart. Nevertheless, according to the ISRII experts, public dissemination of internet programs in different contexts is also highly valued [43]. Indeed, adapting the structure of the mHeart system to other population needs in the same health institution was an aid for recuperating the initial cost and implementation burden. Likewise, other institutions could profit from an already established clinically tested software as a starting point to avoid the burden of developing systems from scratch. An example is how the mHealthCare System, designed as a basis to develop mHeart, has been scaled to different populations by other health care centers (ie, MedPlan+, e-OncoSalud, ePrematur, Entrena EII, Gerar, RC Rehabilitación Cardiaca, and ICOnnecta, among others). Thus, any new upgrade on these apps improves the basis of the software and benefits several institutions.

The implementation of behavioral change technology models targeting complex populations demands a multidisciplinary approach to obtain the strategy benefit [37,51,115]. Operating this process was a highly demanding task, requiring managerial and coordinator profiles with certain skills. The leadership of mHeart implementation by a clinical pharmacist provided a strong opportunity to expand this role into the cardiology team, while making this provider visible to patients, families, and institutions. Likewise, eHealth has resulted in a valuable opportunity to expand the benefits of patient counseling and therapeutic drug monitoring by a multidisciplinary team in health care systems [37,117].

To scale any intervention model into research studies, and in line with the ISRII [43] and the CONSORT-EHEALTH reporting guidelines [47], it is vital to include an in-depth description of the strategy design. Thus, the theoretical framework, mode of delivery, and components of the intervention have been detailed for mHeart. Thereby, a behavioral-based design was used given the potential for providing a better understanding of how the intervention works on patient behaviors [118]. This has in turn been shown to increase efficacy, comparability, and scalability of the interventions performed [43,47]. Based on this background, a pilot study was performed to validate the mHeart tool to improve medication adherence in heart transplant patients. This exploratory study showed that the multilevel behavioral change intervention established (ie, the mHeart strategy) was highly effective since the improvement in adherence to immunosuppressive medication was 30%. Moreover, patient overall satisfaction with the mHeart approach was 9 (on a scale of 0-10) and the mHeart approach demonstrated its potential to overcome the limitations of traditional on-site methods [119]. Based on this experience and in line with other authors [25,70,120,121], it is highly recommended for future studies inspired on the mHeart model to count on providers properly trained in behavioral skills (eg, motivational interviewing) to deliver such theory-based interventions.

### Limitations

This study has some limitations. First, we did not address the efficacy and sustainability of the mHeart approach over time, since the focus of the study was on the model implementation and scalability phases. Therefore, clinical applications of the mHeart strategy will provide information on the impact of its features on health outcomes. In future research conducted with this model, details should be provided by health providers on when and under what conditions interventions will be delivered [54]. Second, based on ISRII recommendations [43], the validity of the electronic versions of the questionnaires used to measure diverse health domains in the mHeart system should also be evaluated before scaling up for larger research. The mHeart electronic questionnaires used to measure medication adherence have been validated and were proven to be as effective as the traditional on-site method in identifying nonadherent recipients in a pilot study [119]. This finding supports their widespread application in larger research and clinical practice. Third, in-depth analysis of the external validation was needed. In this regard, and to support the quality content of the mHeart platform, we obtained external feedback from patients’ representatives of support groups from other centers and institutional endorsement by scientific societies related to the population field. Moreover, the mHeart validation study was also performed to compare the electronic mHeart approach versus the traditional (in-clinic) method to detect nonadherent heart transplant recipients and to improve medication adherence rates [119].

### Conclusions

The experience gained during mHeart implementation has identified the facilitators and key strategies needed for success in new holistic theory–based internet models. It is recommended that future developers direct efforts to verify the experience of the technical team; ensure data confidentiality; and overcome workload, the digital divide, and interoperability. Heart transplant recipients’ access to technology and willingness to use an mHealth approach were confirmed. An interdisciplinary team and a patient-centered design were vital to achieving a comprehensive mHealth approach directed to improve therapy management, patient empowerment, and patient-provider interactions. The mHeart model will be widely applicable in distinct clinical contexts, and may inspire other health providers to create innovative ways to deal with therapeutic complexity and multimorbidity in complex populations.

## Appendix

Multimedia Appendix 1 Behavior change techniques designed to improve patients’ medication and lifestyle habits, adapted to be delivered using the mHeart platform in interventional studies.

Multimedia Appendix 2 Questionnaires and surveys designed to asses participants' data.

Multimedia Appendix 3 Main areas for improvement in mHeart prototype 1 as a result of user feedback during alpha testing.

## Abbreviations

ISRII: International Society for Research on Internet Interventions
mHealth: mobile health

## References

[1] Matesanz R, Domínguez-Gil B, Coll E, Mahíllo B, Marazuela R (2017). How Spain Reached 40 Deceased Organ Donors per Million Population. Am J Transplant.

[2] Wilhelm MJ (2015). Long-term outcome following heart transplantation: current perspective. J Thorac Dis.

[3] González-Vílchez F, Almenar-Bonet L, Crespo-Leiro MG, Alonso-Pulpón L, González-Costelo J, Sobrino-Márquez JM, Arizón del Prado JM, Sousa-Casasnovas I, Delgado-Jiménez J, Pérez-Villa F (2018). Registro Español de Trasplante Cardiaco. XXIX Informe Oficial de la Sección de Insuficiencia Cardiaca de la Sociedad Española de Cardiología (1984-2017). Rev Español Cardiol.

[4] Lund LH, Khush KK, Cherikh WS, Goldfarb S, Kucheryavaya AY, Levvey BJ, Meiser B, Rossano JW, Chambers DC, Yusen RD, Stehlik J, International Society for HeartLung Transplantation (2017). The Registry of the International Society for Heart and Lung Transplantation: Thirty-fourth Adult Heart Transplantation Report-2017; Focus Theme: Allograft ischemic time. J Heart Lung Transplant.

[5] Mercer S, Furler J, Moffat K, Fischbacher-Smith D, Sanci L (2016). Multimorbidity: Technical Series on Safer Primary Care.

[6] Kernick D, Chew-Graham CA, O'Flynn N (2017). Clinical assessment and management of multimorbidity: NICE guideline. Br J Gen Pract.

[7] Lindenfeld J, Page RL, Zolty R, Shakar SF, Levi M, Lowes B, Wolfel EE, Miller GG (2005). Drug therapy in the heart transplant recipient: Part III: common medical problems. Circulation.

[8] Bryant BM, Libby AM, Metz KR, Page RL, Ambardekar AV, Lindenfeld J, Aquilante CL (2016). Evaluating Patient-Level Medication Regimen Complexity Over Time in Heart Transplant Recipients. Ann Pharmacother.

[9] Gomis-Pastor M, Roig Mingell E, Mirabet Perez S, Brossa Loidi V, Lopez Lopez L, Diaz Bassons A, Aretio Pousa A, Feliu Ribera A, Ferrero-Gregori A, Guirado Perich L, Mangues Bafalluy MA (2019). Multimorbidity and medication complexity: New challenges in heart transplantation. Clin Transplant.

[10] De Geest S, Dobbels F, Fluri C, Paris W, Troosters T (2005). Adherence to the therapeutic regimen in heart, lung, and heart-lung transplant recipients. J Cardiovasc Nurs.

[11] Brocks Y, Zittermann A, Grisse D, Schmid-Ott G, Stock-Gießendanner S, Schulz U, Brakhage J, Benkler A, Gummert J, Tigges-Limmer K (2017). Adherence of Heart Transplant Recipients to Prescribed Medication and Recommended Lifestyle Habits. Prog Transplant.

[12] Denhaerynck K, Berben L, Dobbels F, Russell CL, Crespo-Leiro MG, Poncelet AJ, De Geest S, BRIGHT study team (2018). Multilevel factors are associated with immunosuppressant nonadherence in heart transplant recipients: The international BRIGHT study. Am J Transplant.

[13] Korb-Savoldelli V, Sabatier B, Gillaizeau F, Guillemain R, Prognon P, Bégué D, Durieux P (2010). Non-adherence with drug treatment after heart or lung transplantation in adults: a systematic review. Patient Educ Couns.

[14] Kamila P, Smith SG, Patzer R, Wolf MS, Marina S (2014). Medication regimen complexity in kidney and liver transplant recipients. Transplantation.

[15] Libby AM, Fish DN, Hosokawa PW, Linnebur SA, Metz KR, Nair KV, Saseen JJ, Vande Griend JP, Vu SP, Hirsch JD (2013). Patient-level medication regimen complexity across populations with chronic disease. Clin Ther.

[16] Pantuzza LL, Ceccato MDGB, Silveira MR, Junqueira LMR, Reis AMM (2017). Association between medication regimen complexity and pharmacotherapy adherence: a systematic review. Eur J Clin Pharmacol.

[17] Schoonover H, Corbett CF, Weeks DL, Willson MN, Setter SM (2014). Predicting potential postdischarge adverse drug events and 30-day unplanned hospital readmissions from medication regimen complexity. J Patient Saf.

[18] Sevilla-Sanchez D, Molist-Brunet N, Amblàs-Novellas J, Roura-Poch P, Espaulella-Panicot J, Codina-Jané C (2017). Adverse drug events in patients with advanced chronic conditions who have a prognosis of limited life expectancy at hospital admission. Eur J Clin Pharmacol.

[19] Menditto E, Gimeno Miguel A, Moreno Juste A, Poblador Plou B, Aza Pascual-Salcedo M, Orlando V, González Rubio F, Prados Torres A (2019). Patterns of multimorbidity and polypharmacy in young and adult population: Systematic associations among chronic diseases and drugs using factor analysis. PLoS One.

[20] Montiel-Luque A, Núñez-Montenegro AJ, Martín-Aurioles E, Canca-Sánchez JC, Toro-Toro MC, González-Correa JA, Polipresact Research Group (2017). Medication-related factors associated with health-related quality of life in patients older than 65 years with polypharmacy. PLoS One.

[21] Ghimire S, Peterson GM, Castelino RL, Jose MD, Zaidi STR (2016). Medication Regimen Complexity and Adherence in Haemodialysis Patients: An Exploratory Study. Am J Nephrol.

[22] Wimmer BC, Bell JS, Fastbom J, Wiese MD, Johnell K (2016). Medication Regimen Complexity and Number of Medications as Factors Associated With Unplanned Hospitalizations in Older People: A Population-based Cohort Study. J Gerontol A Biol Sci Med Sci.

[23] Wimmer BC, Bell JS, Fastbom J, Wiese MD, Johnell K (2016). Medication Regimen Complexity and Polypharmacy as Factors Associated With All-Cause Mortality in Older People: A Population-Based Cohort Study. Ann Pharmacother.

[24] Dobbels F, De Geest S, van Cleemput J, Droogne W, Vanhaecke J (2004). Effect of late medication non-compliance on outcome after heart transplantation: a 5-year follow-up. J Heart Lung Transplant.

[25] Senft Y, Kirsch M, Denhaerynck K, Dobbels F, Helmy R, Russell CL, Berben L, De Geest S, BRIGHT study team (2018). Practice patterns to improve pre and post-transplant medication adherence in heart transplant centres: a secondary data analysis of the international BRIGHT study. Eur J Cardiovasc Nurs.

[26] Fleming JN, Taber DJ, McElligott J, McGillicuddy JW, Treiber F (2017). Mobile Health in Solid Organ Transplant: The Time Is Now. Am J Transplant.

[27] Karam V, Sebagh M, Rifai K, Yilmaz F, Bhangui P, Danet C, Saliba F, Samuel D, Castaing D, Adam R, Feray C (2016). Quality of life 10 years after liver transplantation: The impact of graft histology. World J Transplant.

[28] Damery S, Flanagan S, Combes G (2015). The effectiveness of interventions to achieve co-ordinated multidisciplinary care and reduce hospital use for people with chronic diseases: study protocol for a systematic review of reviews. Syst Rev.

[29] Damery S, Flanagan S, Combes G (2016). Does integrated care reduce hospital activity for patients with chronic diseases? An umbrella review of systematic reviews. BMJ Open.

[30] Cajita MI, Baumgartner E, Berben L, Denhaerynck K, Helmy R, Schönfeld S, Berger G, Vetter C, Dobbels F, Russell CL, De Geest S, BRIGHT Study Team (2017). Heart transplant centers with multidisciplinary team show a higher level of chronic illness management - Findings from the International BRIGHT Study. Heart Lung.

[31] Costanzo MR, Dipchand A, Starling R, Anderson A, Chan M, Desai S, Fedson S, Fisher P, Gonzales-Stawinski G, Martinelli L, McGiffin D, Smith J, Taylor D, Meiser B, Webber S, Baran D, Carboni M, Dengler T, Feldman D, Frigerio M, Kfoury A, Kim D, Kobashigawa J, Shullo M, Stehlik J, Teuteberg J, Uber P, Zuckermann A, Hunt S, Burch M, Bhat G, Canter C, Chinnock R, Crespo-Leiro M, Delgado R, Dobbels F, Grady K, Kao W, Lamour J, Parry G, Patel J, Pini D, Towbin J, Wolfel G, Delgado D, Eisen H, Goldberg L, Hosenpud J, Johnson M, Keogh A, Lewis C, O'Connell J, Rogers J, Ross H, Russell S, Vanhaecke J, International Society of HeartLung Transplantation Guidelines (2010). The International Society of Heart and Lung Transplantation Guidelines for the care of heart transplant recipients. J Heart Lung Transplant.

[32] Comín-Colet J, Enjuanes C, Verdú-Rotellar JM, Linas A, Ruiz-Rodriguez P, González-Robledo G, Farré N, Moliner-Borja P, Ruiz-Bustillo S, Bruguera J (2016). Impact on clinical events and healthcare costs of adding telemedicine to multidisciplinary disease management programmes for heart failure: Results of a randomized controlled trial. J Telemed Telecare.

[33] Melchiorre MG, Lamura G, Barbabella F, ICARE4EU Consortium (2018). eHealth for people with multimorbidity: Results from the ICARE4EU project and insights from the "10 e's" by Gunther Eysenbach. PLoS One.

[34] Martínez-Pérez B, de la Torre-Díez I, López-Coronado M, Herreros-González J (2013). Mobile apps in cardiology: review. JMIR Mhealth Uhealth.

[35] Kohl LFM, Crutzen R, de Vries NK (2013). Online prevention aimed at lifestyle behaviors: a systematic review of reviews. J Med Internet Res.

[36] Lancaster K, Abuzour A, Khaira M, Mathers A, Chan A, Bui V, Lok A, Thabane L, Dolovich L (2018). The Use and Effects of Electronic Health Tools for Patient Self-Monitoring and Reporting of Outcomes Following Medication Use: Systematic Review. J Med Internet Res.

[37] Alexander E, Butler C, Darr A, Jenkins M, Long R, Shipman C, Stratton T (2017). ASHP Statement on Telepharmacy. Am J Health Syst Pharm.

[38] Littauer SL, Dixon DL, Mishra VK, Sisson EM, Salgado TM (2017). Pharmacists providing care in the outpatient setting through telemedicine models: a narrative review. Pharm Pract (Granada).

[39] Vallespin B, Cornet J, Kotzeva A (2016). Ensuring Evidence-Based Safe and Effective mHealth Applications. Stud Health Technol Inform.

[40] Basch E, Deal AM, Kris MG, Scher HI, Hudis CA, Sabbatini P, Rogak L, Bennett AV, Dueck AC, Atkinson TM, Chou JF, Dulko D, Sit L, Barz A, Novotny P, Fruscione M, Sloan JA, Schrag D (2016). Symptom Monitoring With Patient-Reported Outcomes During Routine Cancer Treatment: A Randomized Controlled Trial. J Clin Oncol.

[41] Park LG, Howie-Esquivel J, Dracup K (2014). A quantitative systematic review of the efficacy of mobile phone interventions to improve medication adherence. J Adv Nurs.

[42] Piette JD, List J, Rana GK, Townsend W, Striplin D, Heisler M (2015). Mobile Health Devices as Tools for Worldwide Cardiovascular Risk Reduction and Disease Management. Circulation.

[43] Ritterband LM, Andersson G, Christensen HM, Carlbring P, Cuijpers P (2006). Directions for the International Society for Research on Internet Interventions (ISRII). J Med Internet Res.

[44] Webb TL, Joseph J, Yardley L, Michie S (2010). Using the internet to promote health behavior change: a systematic review and meta-analysis of the impact of theoretical basis, use of behavior change techniques, and mode of delivery on efficacy. J Med Internet Res.

[45] Heron KE, Smyth JM (2010). Ecological momentary interventions: incorporating mobile technology into psychosocial and health behaviour treatments. Br J Health Psychol.

[46] Free C, Phillips G, Galli L, Watson L, Felix L, Edwards P, Patel V, Haines A (2013). The effectiveness of mobile-health technology-based health behaviour change or disease management interventions for health care consumers: a systematic review. PLoS Med.

[47] Eysenbach G, CONSORT-EHEALTH Group (2011). CONSORT-EHEALTH: improving and standardizing evaluation reports of Web-based and mobile health interventions. J Med Internet Res.

[48] Davidson T, McGillicuddy J, Mueller M, Brunner-Jackson B, Favella A, Anderson A, Torres M, Ruggiero K, Treiber F (2015). Evaluation of an mHealth Medication Regimen Self-Management Program for African American and Hispanic Uncontrolled Hypertensives. J Pers Med.

[49] DeVito Dabbs A, Song MK, Myers BA, Li R, Hawkins RP, Pilewski JM, Bermudez CA, Aubrecht J, Begey A, Connolly M, Alrawashdeh M, Dew MA (2016). A Randomized Controlled Trial of a Mobile Health Intervention to Promote Self-Management After Lung Transplantation. Am J Transplant.

[50] Rosenberger EM, DeVito Dabbs AJ, DiMartini AF, Landsittel DP, Pilewski JM, Dew MA (2017). Long-Term Follow-up of a Randomized Controlled Trial Evaluating a Mobile Health Intervention for Self-Management in Lung Transplant Recipients. Am J Transplant.

[51] Poudel A, Nissen L (2016). Integr Pharm Res Pract.

[52] Pinnock H, Barwick M, Carpenter CR, Eldridge S, Grandes G, Griffiths CJ, Rycroft-Malone J, Meissner P, Murray E, Patel A, Sheikh A, Taylor SJC, StaRI Group (2017). Standards for Reporting Implementation Studies (StaRI) Statement. BMJ.

[53] Tong A, Sainsbury P, Craig J (2007). Consolidated criteria for reporting qualitative research (COREQ): a 32-item checklist for interviews and focus groups. Int J Qual Health Care.

[54] Mohr DC, Schueller SM, Montague E, Burns MN, Rashidi P (2014). The behavioral intervention technology model: an integrated conceptual and technological framework for eHealth and mHealth interventions. J Med Internet Res.

[55] Ritterband LM, Thorndike FP, Cox DJ, Kovatchev BP, Gonder-Frederick LA (2009). A behavior change model for internet interventions. Ann Behav Med.

[56] Oinas-Kukkonen H (2012). A foundation for the study of behavior change support systems. Pers Ubiquit Comput.

[57] Oinas-Kukkonen H, Harjumaa M (2009). Persuasive Systems Design: Key Issues, Process Model, and System Features. Commun Assoc Inf Syst.

[58] Fogg BJ (2009). A behavior model for persuasive design.

[59] Whittaker R, Merry S, Dorey E, Maddison R (2012). A development and evaluation process for mHealth interventions: examples from New Zealand. J Health Commun.

[60] Yen P, Bakken S (2012). Review of health information technology usability study methodologies. J Am Med Inform Assoc.

[61] Kelders SM, Kok RN, Ossebaard HC, Van Gemert-Pijnen JEWC (2012). Persuasive system design does matter: a systematic review of adherence to web-based interventions. J Med Internet Res.

[62] Baumel A, Kane JM (2018). Examining Predictors of Real-World User Engagement with Self-Guided eHealth Interventions: Analysis of Mobile Apps and Websites Using a Novel Dataset. J Med Internet Res.

[63] Pagès-Puigdemont N, Mangues MA, Masip M, Gabriele G, Fernández-Maldonado L, Blancafort S, Tuneu L (2016). Patients' Perspective of Medication Adherence in Chronic Conditions: A Qualitative Study. Adv Ther.

[64] Davis R, Campbell R, Hildon Z, Hobbs L, Michie S (2015). Theories of behaviour and behaviour change across the social and behavioural sciences: a scoping review. Health Psychol Rev.

[65] Conn VS, Enriquez M, Ruppar TM, Chan KC (2016). Meta-analyses of Theory Use in Medication Adherence Intervention Research. Am J Health Behav.

[66] Kok G, Schaalma H, Ruiter RAC, van Empelen P, Brug J (2004). Intervention mapping: protocol for applying health psychology theory to prevention programmes. J Health Psychol.

[67] Michie S, Johnston M, Francis J, Hardeman W, Eccles M (2008). From Theory to Intervention: Mapping Theoretically Derived Behavioural Determinants to Behaviour Change Techniques. Appl Psychol.

[68] Abraham C, Michie S (2008). A taxonomy of behavior change techniques used in interventions. Health Psychol.

[69] Muench F, Baumel A (2017). More Than a Text Message: Dismantling Digital Triggers to Curate Behavior Change in Patient-Centered Health Interventions. J Med Internet Res.

[70] Dobbels F, De Bleser L, Berben L, Kristanto P, Dupont L, Nevens F, Vanhaecke J, Verleden G, De Geest S (2017). Efficacy of a medication adherence enhancing intervention in transplantation: The MAESTRO-Tx trial. J Heart Lung Transplant.

[71] Kay M, World Health Organization, Global Observatory for eHealth (2011). mHealth: New horizons for health through mobile technologies. Global Observatory for eHealth series: Volume 3.

[72] (2014). Green Paper on mobile health (“mHealth”).

[73] (2013). Socio-economic impact of mHealth. An assessment report for the European Union. Pricewaterhouse Coopers Private Limited.

[74] (2019). Policy for Device Software Functions and Mobile Medical Applications. Guidance for Industry and Food and Drug Administration Staff.

[75] Cortez NG, Cohen IG, Kesselheim AS (2014). FDA regulation of mobile health technologies. N Engl J Med.

[76] (2012). eHealth Action Plan 2012-2020 -Innovative healthcare for the 21st century Internet. European Commission.

[77] PLOS Medicine Editors (2013). A reality checkpoint for mobile health: three challenges to overcome. PLoS Med.

[78] Hamine S, Gerth-Guyette E, Faulx D, Green BB, Ginsburg AS (2015). Impact of mHealth chronic disease management on treatment adherence and patient outcomes: a systematic review. J Med Internet Res.

[79] McGillicuddy JW, Gregoski MJ, Weiland AK, Rock RA, Brunner-Jackson BM, Patel SK, Thomas BS, Taber DJ, Chavin KD, Baliga PK, Treiber FA (2013). Mobile Health Medication Adherence and Blood Pressure Control in Renal Transplant Recipients: A Proof-of-Concept Randomized Controlled Trial. JMIR Res Protoc.

[80] Rao S, Ghanta M, Moritz MJ, Constantinescu S (2016). Long-Term Functional Recovery, Quality of Life, and Pregnancy After Solid Organ Transplantation. Med Clin North Am.

[81] Dew MA, Goycoolea JM, Harris RC, Lee A, Zomak R, Dunbar-Jacob J, Rotondi A, Griffith BP, Kormos RL (2004). An internet-based intervention to improve psychosocial outcomes in heart transplant recipients and family caregivers: development and evaluation. J Heart Lung Transplant.

[82] Berben L, Denhaerynck K, Dobbels F, Engberg S, Vanhaecke J, Crespo-Leiro MG, Russell CL, De Geest S, BRIGHT study consortium (2015). Building research initiative group: chronic illness management and adherence in transplantation (BRIGHT) study: study protocol. J Adv Nurs.

[83] Hugon A, Roustit M, Lehmann A, Saint-Raymond C, Borrel E, Hilleret M, Malvezzi P, Bedouch P, Pansu P, Allenet B (2014). Influence of intention to adhere, beliefs and satisfaction about medicines on adherence in solid organ transplant recipients. Transplantation.

[84] Kobashigawa J, Zuckermann A, Macdonald P, Leprince P, Esmailian F, Luu M, Mancini D, Patel J, Razi R, Reichenspurner H, Russell S, Segovia J, Smedira N, Stehlik J, Wagner F, Consensus Conference participants (2014). Report from a consensus conference on primary graft dysfunction after cardiac transplantation. J Heart Lung Transplant.

[85] Lee JL, Eaton C, Gutiérrez-Colina AM, Devine K, Simons LE, Mee L, Blount RL (2014). Longitudinal stability of specific barriers to medication adherence. J Pediatr Psychol.

[86] McGillicuddy J, Weiland A, Frenzel R, Mueller M, Brunner-Jackson B, Taber D, Baliga P, Treiber F (2013). Patient attitudes toward mobile phone-based health monitoring: questionnaire study among kidney transplant recipients. J Med Internet Res.

[87] Viladrich C, Doval E (2011). Medición: Fiabilidad y Validez. 5a ed Bellaterra: Laboratori d'estadística Aplicada i Modelització.

[88] Gomis-Pastor M, Mangues M, Pellicer V (2019). mHeart - mHealthCare Platform Adapted to the Heart Transplant Population - Technical Specifications and Software Source Code Internet. Mendeley Data.

[89] Clinapsis: Clinical Epidemiology and Healthcare Services.

[90] (2019). mHealthCare Platform Trilema Salud Internet. Fundación Trilema.

[91] mHeart - Google Play Mobile Applications Internet. Socioemprende SL (TrilemaSalud Group).

[92] mHeart - App Store Mobile Applications Internet. Socioemprende S.L (TrilemaSalud Group).

[93] Sackett DL, Haynes RB, Fuyatt GH, Tungwell P (1994). Ayudar a los pacientes a cumplir los tratamientos. Epidemiol clínica, Cienc básica para la Med clínica 2a ed.

[94] Carbonell Abella C, Guañabens Gay N, Regadera Anechina L, Marín Rives JA, Taverna Llauradó E, Ayechu Redín MP, ADHEPOR (2011). [Analysis of therapeutic compliance in women with osteoporosis]. Reumatol Clin.

[95] Fernandez-Mendoza J, Rodriguez-Muñoz A, Vela-Bueno A, Olavarrieta-Bernardino S, Calhoun SL, Bixler EO, Vgontzas AN (2012). The Spanish version of the Insomnia Severity Index: a confirmatory factor analysis. Sleep Med.

[96] Rabin R, de Charro F (2001). EQ-5D: a measure of health status from the EuroQol Group. Ann Med.

[97] (2019). Regulatory framework: Internal Market, Industry, Entrepreneurship and SMEs Internet. European Commission.

[98] (2012). AppSaludable Quality Seal Internet. Agencia de Calidad Sanitaria de Andalucía.

[99] Guidebooks for the accreditation process for health or wellness Apps. Fundació TIC Salut Social. Generalitat de Catalunya.

[100] Behaviour change: general approaches (PH6). National Institute for Health and Care Excellence (NICE).

[101] McMillan B, Hickey E, Patel MG, Mitchell C (2016). Quality assessment of a sample of mobile app-based health behavior change interventions using a tool based on the National Institute of Health and Care Excellence behavior change guidance. Patient Educ Couns.

[102] Inscripción TOP20 Internet. iSYS Foundation.

[103] Stoyanov SR, Hides L, Kavanagh DJ, Wilson H (2016). Development and Validation of the User Version of the Mobile Application Rating Scale (uMARS). JMIR Mhealth Uhealth.

[104] Domnich A, Arata L, Amicizia D, Signori A, Patrick B, Stoyanov S, Hides L, Gasparini R, Panatto D (2016). Development and validation of the Italian version of the Mobile Application Rating Scale and its generalisability to apps targeting primary prevention. BMC Med Inform Decis Mak.

[105] Williams A, Low JK, Manias E, Dooley M, Crawford K (2016). Trials and tribulations with electronic medication adherence monitoring in kidney transplantation. Res Social Adm Pharm.

[106] (2018). A quick guide to recommendations for the development of health and social care Apps. How to create an application secure, accessible mobile usable and interoperable. Fundació TIC Salut Social. Generalitat de Catalunya.

[107] Summary report on public consultation on the green paper on mobile health. European Comission.

[108] Israni A, Dean C, Kasel B, Berndt L, Wildebush W, Wang CJ (2016). Why do Patients Forget to Take Immunosuppression Medications and Miss Appointments: Can a Mobile Phone App Help?. JMIR Public Health Surveill.

[109] Perski O, Blandford A, West R, Michie S (2017). Conceptualising engagement with digital behaviour change interventions: a systematic review using principles from critical interpretive synthesis. Transl Behav Med.

[110] Yardley L, Spring BJ, Riper H, Morrison LG, Crane DH, Curtis K, Merchant GC, Naughton F, Blandford A (2016). Understanding and Promoting Effective Engagement With Digital Behavior Change Interventions. Am J Prev Med.

[111] Molokhia M, Majeed A (2017). Current and future perspectives on the management of polypharmacy. BMC Fam Pract.

[112] Kim DH, Rich MW (2016). Patient-Centred Care of Older Adults With Cardiovascular Disease and Multiple Chronic Conditions. Can J Cardiol.

[113] García-Jiménez E, Amariles P, González M, Parras-Martín M, Espejo-Guerrero J, Faus M (2008). Non-adherence, drug-related problems and negative outcomes associated with medication: Causes and outcomes in drug therapy follow-up. Ars Pharm.

[114] Sankaranarayanan J, Murante LJ, Moffett LM (2014). A retrospective evaluation of remote pharmacist interventions in a telepharmacy service model using a conceptual framework. Telemed J E Health.

[115] Niznik JD, He H, Kane-Gill SL (2018). Impact of clinical pharmacist services delivered via telemedicine in the outpatient or ambulatory care setting: A systematic review. Res Social Adm Pharm.

[116] Checchi KD, Huybrechts KF, Avorn J, Kesselheim AS (2014). Electronic medication packaging devices and medication adherence: a systematic review. JAMA.

[117] Strand M, Tellers J, Patterson A, Ross A, Palombi L (2016). The achievement of public health services in pharmacy practice: A literature review. Res Social Adm Pharm.

[118] Michie S, Wood CE, Johnston M, Abraham C, Francis JJ, Hardeman W (2015). Behaviour change techniques: the development and evaluation of a taxonomic method for reporting and describing behaviour change interventions (a suite of five studies involving consensus methods, randomised controlled trials and analysis of qualitative data). Health Technol Assess.

[119] Gomis-Pastor Mar, Roig Eulalia, Mirabet Sonia, T De Pourcq Jan, Conejo Irene, Feliu A, Brossa Vicens, Lopez Laura, Ferrero-Gregori Andreu, Barata Anna, Mangues M Antonia (2020). A Mobile App (mHeart) to Detect Medication Nonadherence in the Heart Transplant Population: Validation Study. JMIR Mhealth Uhealth.

[120] Salvo MC, Cannon-Breland ML (2015). Motivational interviewing for medication adherence. J Am Pharm Assoc (2003).

[121] Morton K, Beauchamp M, Prothero A, Joyce L, Saunders L, Spencer-Bowdage S, Dancy B, Pedlar C (2015). The effectiveness of motivational interviewing for health behaviour change in primary care settings: a systematic review. Health Psychol Rev.

