# Supplementary material for: Interdisciplinary Mobile Health Model to Improve Clinical Care After Heart Transplantation: Implementation Strategy Study
Source: JMIR Cardio. 2020 Nov 24;4(1):e19065. doi: 10.2196/19065 (PMC7723747; doi:10.2196/19065)
Supplement: Multimedia Appendix 1 [file cardio_v4i1e19065_app1.pdf]

Multimedia Appendix 1

Behavior change techniques designed to improve patients' medication and lifestyle habits and adapted for be delivered using the mHeart platform in interventional studies.

Along with the technique and the theoretical framework, a description is provided to facilitate the selection of the adequate technique according to the clinical aim to be achieved.

[a]Technique (Theory)	Description of the theory-based intervention technique in mHeart
Motivational communication skills (MI)	Use of any patient- provider communication opportunity to prompt the patient to provide self-motivating statements and self-evaluations: (1) minimize resistance to change; (2) maintain the change achieved.
Tailoring	Individualize the interventions provided based on the patient's environment and self-reports.
Provide instructions (SCogT)	Tell the patient how to adopt a health-related behavior, e.g. providers' direct message alerting patients of a prescription change and how to take it.
Time management	Find a timetable for drug intakes and lifestyle habits that fits with each patient's routine.
Goal setting (CT)	Plan together with patients the steps to acquire the skills needed for the new health habit (specifying frequency, intensity, or duration and context).
Provide information (IMB, CT)	Provide information on the behavior-health links and consequences by using the individual or mass campaigns of the mHeart messaging system.
Prompt behavior self-monitoring (CT)	Ask the patient to report data related to distinct behaviors, e.g. drug intakes, side effects, blood pressure, electronic questionnaires, among others PRO[c].
Provide feedback on performance (CT)	Provide feedback by messaging or in-clinics visit based on the self-reported information to maintain patient enhance with the intervention program.
Provide contingent rewards (OC)	Provide praise or encouragement linked to the achievement of specified behaviors, e.g. praise any improvement in self-efficacy.
Prompt review of behavioral goals (CT)	Review periodically the intentions or goals previously agreed with the patient. Discuss and readjust the plan if necessary.

Identify barriers to behavior (SCogT)	Identify the barriers to adequate implementation of a new behavior using patient-reported outcome measures[c], e.g. detect a side effect which is curving adherence to medication.
Action planning & problem solving	Plan ways of overcoming the barriers detected and reach an agreement with the patient, e.g. discuss medication beliefs with the patient.
Environmental restructuring	Provide guidance to change the patient's habits that could hamper the medication or lifestyle behavior.
Teaching the use of prompts/cues (OC)	Teach patients to identify environmental cues to remind them to adopt a behavior.
Prompt intention formation (TRA, TPB, SCogT, IMB)	Encourage the patient to decide to act or set a general goal by making a behavioral resolution, e.g. "I will take my pills on time every day".
Prompting focus on past success	Discuss or review with the patient past behaviors related to negative outcomes.
Others' approval (TRA, TPB, IMB)	Provide information on what others think of a behavior, e.g. inform the patient that providers will disapprove of an unhealthy behavior.
Provide information on others' behavior	Compare anonymous experiences to encourage or reduce the patient's feeling of burden.
Use follow-up prompts	Send messages to the patient after a part of the challenge has been completed. Especially when (1) several goals were planned; (2) a behavior has changed gradually.
Prompt identification as a role model	Indicate how the patient may be an example to others and influence their behavior.

[a] Techniques inspired by the Abraham and Michie's taxonomy (2008). The theoretical frameworks are: CT, control theory; IMB, information-motivation-behavioral skills model; MI, Motivational Interview; OC, operant conditioning; SCogT, social-cognitive theory; TPB, theory of planned behavior; TRA, theory of reasoned action.
