# Supplementary material for: Interdisciplinary Mobile Health Model to Improve Clinical Care After Heart Transplantation: Implementation Strategy Study
Source: JMIR Cardio. 2020 Nov 24;4(1):e19065. doi: 10.2196/19065 (PMC7723747; doi:10.2196/19065)
Supplement: Multimedia Appendix 2 [file cardio_v4i1e19065_app2.pdf]

Multimedia Appendix 2

Questionnaires and surveys designed to asses participants data.

2.1.Questionnaire designed to perform prospective face-to-face interviews to heart transplant recipients. Including sociodemographic and clinical data, but also technology use and willingness

Has the patient signed the informed consent? Yes<br>Has the patient fulfilled the inclusion criteria? Yes	Interview date by MG: __/__/____<br>Prospective review of the patient EHR: __/__/____
**Sociodemographic and clinical variables**	
Patient Hospital's code: _________<br>Date of birth: __/__/____<br>Gender<br>☐ Male<br>☐ Female<br>☐ Other<br>Weight: ___ kilograms<br>Height: ___ meters	Date of the heart transplant: __/__/___<br>Urgent heart transplant:<br>☐ Yes<br>☐ No<br>Heart failure etiology:<br>☐ Congenital<br>☐ Coronary/ischemic<br>☐ Myocarditis<br>☐ Cardiomyopathy<br>☐ Valvular cardiac disease<br>☐ Hypertrophic cardiomyopathy<br>☐ Re-transplant<br>☐ Other
Educational attainment<br>☐ No formal education<br>☐ Middle school graduate<br>☐ High school graduate<br>☐ University graduate	Employment status:<br>☐ Incapacity<br>☐ Retired<br>☐ No previous employment<br>☐ Currently employed
Need or requirement for caregiver<br>☐ Yes<br>☐ No	Lives with someone else<br>☐ Yes<br>☐ No
Number of comorbidities (EHR and confirmed by the patient): ____	
Type of comorbidity post-transplant (multiple choice):<br>☐ High blood pressure<br>☐ Dyslipidemia<br>☐ Chronic kidney failure<br>☐ Osteopathies and chondroplasties<br>☐ Diseases of the nervous system<br>☐ Mood and anxiety disorders	☐ Digestive system diseases or disorders<br>☐ Diabetes Mellitus<br>☐ Neoplasia<br>☐ Arthropathies<br>☐ Others: ______________________________<br>______________________________
Total number of drugs prescribed (EHR and confirmed by the patient): ____	
**Technology availability, use and acceptance variables**	
Number of devices per patient: _____	
Types of devices owned by the patient<br>☐ Mobile Phone<br>☐ Computer<br>☐ Tablet	
Type of patient internet connection on their device:<br>☐ 3G or 4G connection<br>☐ Only connects to the internet using the WIFI<br>☐ Does not know/does not answer	
How often does the patient use the technology?<br>☐ Often<br>☐ Sporadically	

☐ Never
Patient use of the internet for health-related purposes:<br>☐ Often<br>☐ Sporadically<br>☐ Never
Do you think it would be useful to use an app or website developed by your transplant team to improve your home-based management and therapy follow-up?<br>☐ Not very useful<br>☐ Useful<br>☐ Very useful<br>☐ Not yet known until the platform is tested
Are you interested in using the mHeart platform by…?<br>☐ A mobile app<br>☐ The website<br>☐ Both (an app and a website)<br>☐ Not yet known until the platform is tested
Do you think that you require personal assistance in using the platform?<br>☐ Yes<br>☐ No<br>☐ Not yet known until the platform is tested

EHR, Electronic Health Records

2.2.Survey designed to assess stakeholder agreement on the benefits and barriers associated with mHealth in multimorbid patients with polypharmacy in our catchment area

Dear Respondent,<br>Thank you very much for accepting our request to participate in this survey. We welcome your honest opinion about the benefits and/or barriers of using an mHealth platform in our catchment area for the follow-up of chronic patients taking multiple medications.<br>Please tick *Yes* or *No* to indicate whether you agree to the following questions and also state the reasons for your answers or any relevant comments you may wish to share with us.<br>Please note that you are welcome to add new topics or ideas not initially included.
**Please complete the following demographic information:**
Date of birth:<br>Gender:<br>Job category:<br>Affiliation:

**Do you agree with the following statements about the benefits of mHealth in the catchment area?**	
1. Improves knowledge of therapy, management and drug adherence in patients at home.<br>*Comments:*	☐ Yes<br>☐ No
2. Improves the continuity of care and the information flow between providers and levels of care.<br>*Comments:*	☐ Yes<br>☐ No
3. Allows for patients to be empowered to actively intervene in managing their disease and treatment.<br>*Comments:*	☐ Yes<br>☐ No
4. Resolves patient and caregiver queries from home due to 2-way clinical-patient communication.<br>*Comments:*	☐ Yes<br>☐ No
5. Allows detection of symptoms and adverse effects to drugs, thereby improving their management.<br>*Comments:*	☐ Yes<br>☐ No
6. Focuses on health promotion and prevention, reducing the number of acute events.<br>*Comments:*	☐ Yes<br>☐ No
7. Increases the cost-effectiveness of resources by reducing scheduled visits, as well as urgent visits due to decompensations.<br>*Comments:*	☐ Yes<br>☐ No
8. Facilitates innovation in health and documentation of evidence that translates into measurable health outcomes.<br>*Comments:*	☐ Yes<br>☐ No
9. Reduces inequalities in access to the health system due to traveling difficulties or lack of resources.<br>*Comments:*	☐ Yes<br>☐ No
10. Improves patients' experience because of close communication with providers<br>*Comments:*	☐ Yes<br>☐ No
Additional comments or suggestions: ______________________<br>______________________<br>______________________	
**Do you agree with the following statements on potential mHealth barriers in the catchment area?**	

1. Increase in workload for staff.<br>*Comments:*	☐ Yes<br>☐ No
2. Lack of institutional guidelines for the development and accreditation of mobile applications in healthcare.<br>*Comments:*	☐ Yes<br>☐ No
3. Risk of not sharing the patient's registered information with other levels of care or with other apps (used to manage other health conditions).<br>*Comments:*	☐ Yes<br>☐ No
4. Risk of not protecting confidential patient data.<br>*Comments:*	☐ Yes<br>☐ No
5. Risk of creating inequalities in patient care due to resistance to use technology or the digital divide [51].<br>*Comments:*	☐ Yes<br>☐ No
6. Lack of guarantees on the long-term economic sustainability of research projects for innovative technologies and companies developing them.<br>*Comments:*	☐ Yes<br>☐ No
Additional comments or suggestions:	

2.3.Questionnaire sent to Regional Health Authorities and the Hospital's Information Analysis Department

Dear Respondent,<br>Thank you very much for accepting our request to participate in this survey.<br>With the overall aim of automatically integrating the new mHeart® platform with patients' sociodemographic and clinical electronic health records (EHR) and to share the data collected by patients in the tool with health providers across levels of care, it is necessary to consider the resources available in the catchment area.<br>For this purpose, please complete this short survey using your own professional expertise.<br>Thank you very much in advance from the Hospital Scientific Advisory Team in charge of the mHeart system.
1. Taking into account the technological resources available, how would you ensure that sociodemographic, clinical and therapeutic patient data is not being manually entered by the providers in the mHeart® prototype?<br>Response (free text):
2. Regarding the sharing of the data collected by patients in the mHeart® platform with health providers across distinct levels of care:<br>a. How could we incorporate patient information collected in the mHeart® platform to the patient's current hospital electronic records (i.e. hospital information system)?<br>Response (free text):<br><br>b. How could we incorporate patient information collected in the mHeart® platform with the electronic records other existing care levels (e.g. primary care)?<br>Response (free text):
