# Supplementary material for: Interdisciplinary Mobile Health Model to Improve Clinical Care After Heart Transplantation: Implementation Strategy Study
Source: JMIR Cardio. 2020 Nov 24;4(1):e19065. doi: 10.2196/19065 (PMC7723747; doi:10.2196/19065)
Supplement: Multimedia Appendix 3 [file cardio_v4i1e19065_app3.pdf]

Multimedia Appendix 3

Main areas for improvement in mHeart Prototype 1 as a result of user feedback during Alpha testing

General Settings	
Patient	- Improve visualization of the menu login button.<br>- Provide direct messaging for contacting the developer's technical team.
Provider	- Adapt the Web to a responsive design.<br>- Modify the user password every 3 months by an automatic message.<br>- Avoid manual input of patient data on discharge.<br>- Supply the legal conditions of use that the patient accepts.<br>- Decrease the number of seconds needed to access the app.<br>- Multi-device access to the patient's app (e.g. if caregivers need to be included).<br>- Include a provider monitoring section from which vital signs registered in follow-up (blood pressure or analytical data) can be consulted and downloaded in Excel format.
Personal and Clinical Data Module	
Patient	- Provide health professional job profiles in the clinical team list. The profile also would be visible when the patient selects the recipient of a message (e.g. Name Surname - Pharmacist).
Provider	- Include a summary heading with each patient's main data, together with a photograph.<br>- Consult the information recorded by a patient in the clinical history available in any center in the health area.
Treatment Module	
Patient	- Consult with the heart transplant team about any incompatibility with courses of treatment prescribed by other providers in the health area.<br>- Facilitate queries referring to other complementary treatments.<br>- Retrospectively validate drug intake and allow multiple validations at the same time.
Provider	- Improve diverse aspects of the drug prescription module to make it quicker and more user-friendly.
Agenda Module	
Patient	- Add photographs of drug packaging to identify it.
Provider	- Change the main screen of the app to the Agenda directly. Change the main screen of the website to the Modules list.<br>- Modify the color of the icons to differentiate the distinct types of notification tasks.<br>- Include another type of reminder on the list: "tests" and "visits".
Patient-Centered Outcomes Module	
Patient	- Include a function to monitor glycemia and temperature.<br>- Improve visualization of the rating of perceived physical exertion (using the Borg Scale).
Provider	- Modify the adherence graph for easier viewing of responses.<br>- Modify the patient-reported outcome data charts to include the average maximum and minimum for the time indicated.<br>- Modify the charts to automatically incorporate data corresponding to the last month.
Symptoms Module	
Provider	- Include email alerts related to extremely serious symptoms notified by patients via the platform and new courses of treatment included by the patient.<br>- Add "vomiting" as a very serious symptom.
Health Education and Advice Module	

Patient	- Provide post-transplant lifestyle and dietary recommendations.
Provider	- Add recommendations and advice using videos.
Teleconsultation and Messaging Module	
Patient	- Enable archives to be uploaded in messages.
Provider	- Permit to send provider campaigns through the messaging system by text-messages in bulk to all patients or to a group of them via filters.
